# Single-Cell and Spatial Transcriptomics Reveals Selenoproteins Shape Immunosuppressive Microenvironment and Therapeutic Outcomes in Glioma

**DOI:** 10.3390/cancers18091489

**Published:** 2026-05-06

**Authors:** Xiaowei Zhang, Na Zhang, Yuqing Zhong, Siqi Ou, Guitao Wu, Taohui Ouyang, Kejun He

**Affiliations:** 1 The First Affiliated Hospital of Sun Yat-sen University, Guangzhou 510062, China; zhangxw93@mail.sysu.edu.cn (X.Z.); zhongyq69@mail.sysu.edu.cn (Y.Z.); ousiqi_zsyy@163.com (S.O.); 2 Department of Neurosurgery, The First Affiliated Hospital of Sun Yat-sen University, Guangzhou 510062, China; 3 Department of Neurology, The First Affiliated Hospital, Jiangxi Medical College, Nanchang University, Nanchang 330006, China; ndyfy04826@ncu.edu.cn; 4 Guangzhou Women and Children’s Hospital, Guangzhou 510623, China; wuguitao@gzfezx.wecom.work; 5 Department of Neurosurgery, The First Affiliated Hospital, Jiangxi Medical College, Nanchang University, Nanchang 330006, China

**Keywords:** selenoproteins, glioblastoma, SELENOS, spatial transcriptomics

## Abstract

Background: Gliomas exhibit substantial intratumoral heterogeneity, which limits prognostic precision and therapeutic efficacy. Selenoproteins are key regulators of redox homeostasis, but their role in glioma progression remains insufficiently defined. This study aimed to characterize glioma cells with high selenoprotein activity and to determine their biological and clinical significance. Methods: We performed integrated multi-omic analyses combining bulk transcriptomic, single-cell transcriptomic, and spatial transcriptomic data to identify and characterize glioma cell states associated with elevated selenoprotein expression. Functional validation was conducted using SELENOS knockdown assays to evaluate effects on glioma proliferation, invasion, tumor growth, macrophage recruitment, CSF1 expression, and macrophage polarization. Results: We identified a malignant glioma cell state, termed SehighMali, characterized by elevated selenoprotein expression and distinct metabolic and immunological features. SehighMali cells showed enhanced oxidative phosphorylation, MYC-associated transcription, and DNA repair activity, and preferentially engaged in immunosuppressive crosstalk with myeloid cells through the CSF1–CSF1R axis. Spatial analyses demonstrated enrichment of SehighMali cells in tumor cores and close colocalization with immunosuppressive myeloid populations. Across bulk cohorts, higher SehighMali abundance was associated with aggressive molecular features, poor clinical outcomes, and a predicted temozolomide-resistant phenotype. SELENOS knockdown suppressed glioma proliferation, invasion, and tumor growth, reduced macrophage recruitment, decreased CSF1 expression, and promoted macrophage polarization toward a pro-inflammatory phenotype. Conclusions: These findings define a selenoprotein-driven malignant glioma state associated with immune evasion and therapeutic vulnerability. They further identify SELENOS as a potential therapeutic target and provide insight into how selenoprotein-related programs contribute to glioma progression.

## Simple Summary

Gliomas are highly heterogeneous brain tumors, and this heterogeneity complicates prognosis and treatment. In this study, we identified a subgroup of glioma cells with high selenoprotein expression, termed SehighMali, which is associated with aggressive tumor features, immune suppression, and poor clinical outcomes. These cells are enriched in tumor core regions and closely associated with immunosuppressive myeloid cells. We further found that SELENOS plays a key role in promoting glioma growth, invasion, and macrophage recruitment. Suppressing SELENOS inhibited tumor progression and shifted macrophages toward a more pro-inflammatory state. These findings improve understanding of how selenoprotein-related programs contribute to glioma progression and suggest SELENOS as a potential therapeutic target.

## 1. Introduction

Gliomas, including diffuse astrocytomas and glioblastomas, are characterized by remarkable cellular heterogeneity and a notoriously poor clinical prognosis [1]. Even with maximal surgery, radiation, and chemotherapy, infiltrative tumor cells almost invariably persist and drive recurrence [2,3]. Both intertumoral and intratumoral heterogeneity enable subsets of glioma cells with the ability to evade treatment, promoting disease progression and relapse [4,5]. Large-scale consortia such as The Cancer Genome Atlas (TCGA) and the Chinese Glioma Genome Atlas (CGGA) have catalogued the genomic and transcriptomic landscapes of gliomas, identifying molecular subtypes and prognostic markers [6,7,8]. However, these bulk tissue analyses do not capture the full spectrum of functional cell states within tumors, nor do they fully explain the metabolic adaptations that glioma cells exploit to survive in the hostile brain microenvironment.

The central nervous system exhibits high oxygen consumption and a lipid-rich composition, predisposing it to elevated oxidative stress [9]. Its relatively limited antioxidant capacity further necessitates tightly regulated redox systems to maintain cellular homeostasis. Selenoproteins are selenocysteine (sec)-containing proteins and are critical for maintaining redox balance and cellular stress tolerance [10,11,12]. While selenoproteins such as GPXs and TXNRDs have been implicated in the progression of various cancers [13,14,15], emerging evidence suggests that specific selenoproteins contribute to glioma malignancy. For example, SELENOS correlates with worse survival in lower-grade gliomas and promotes glioma cell proliferation and invasion in vitro [16]. Silencing of SELENOK suppresses the proliferation of glioma cells [17]. Inhibition of TXNRD1 suppresses GBM cell growth [18]. These findings suggest that selenoproteins may contribute to malignant phenotypes in glioma. However, their functions across distinct cell types, coordinated transcriptional programs, and spatially organized roles within the glioma microenvironment remain essentially unknown. In particular, it is unclear whether selenoproteins define distinct malignant cell states or how they shape metabolic and immune landscapes.

Despite extensive efforts, immunotherapy has shown limited efficacy in glioma. Clinical trials such as CheckMate-143 (anti–PD-1 therapy) and ACT IV (EGFRvIII-targeted vaccination) have failed to significantly improve overall survival in patients with glioblastoma [19,20]. These outcomes are largely attributed to the profoundly immunosuppressive tumor microenvironment, characterized by dysfunctional immune infiltration and dominant myeloid-mediated suppression [21]. Understanding the cellular and molecular mechanisms that shape this immunosuppressive niche therefore remains a critical challenge. Recent advances in single-cell and spatial transcriptomics have enabled high-resolution dissection of glioma ecosystems, revealing remarkable cellular heterogeneity within both malignant and non-malignant compartments [2,5]. Single-cell RNA sequencing (scRNA-seq) uncovers distinct cell populations and novel transcriptional programs at single-cell resolution. In glioblastoma, this approach has identified diverse malignant subpopulations and their dynamic interactions with the tumor microenvironment [5,22]. Spatial transcriptomics complements these findings by preserving the spatial context of gene expression, offering insights into how malignant and stromal cells are organized within the tumor [23,24]. For example, GBmap, a large-scale atlas of glioblastoma, recently delineated spatially defined regions of hypoxia with prognostic significance [25]. These technologies provide a powerful framework for dissecting glioma heterogeneity, metabolism, and therapeutic resistance.

Here, we sought to determine whether selenoproteins define distinct malignant cell states in glioma and how these states integrate into the metabolic and immune architecture of the tumor. Using bulk, single-cell, and spatial transcriptomic data from large glioma cohorts, we identify a transcriptionally distinct malignant cell population referred to as SehighMali, which is characterized by elevated selenoprotein expression. SehighMali cells exhibit enhanced oxidative phosphorylation, MYC pathway activation, and immune-suppressive signaling, and are predominantly enriched in the tumor core. These cells preferentially interact with myeloid populations through CSF1–CSF1R signaling, contributing to the formation of an immunosuppressive tumor microenvironment. The abundance of SehighMali cells correlates with poor clinical outcomes, and predicted resistance to temozolomide, suggesting that this cell state may represent a clinically significant and targetable vulnerability in glioma.

## 2. Materials and Methods

### 2.1. Cell Culture and Transfections

NHA, HS683, SW1783, U251, U87MG, and LN229 cell lines were cultured in a humidified incubator at 37 °C with 5% CO_2_ in Dulbecco’s modified Eagle’s medium (DMEM) supplemented with 10% bovine calf serum. THP-1 cells were cultured in RPMI-1640 medium supplemented with 10% FBS. Differentiation into macrophage-like cells was induced by treatment with 0.1 μg/mL phorbol-12-myristate-13-acetate PMA (Sigma-Aldrich, St. Louis, MO, USA). Lentiviral constructs expressing non-overlapping shRNAs targeting SELENOS (sh1: TRCN0000315839 and sh2: TRCN0000315868), and a non-targeting control shRNA (TRCN0000231489) were acquired from sigma. Lentiviral particles were produced in 293T cells by co-transfecting plasmids with the packaging vectors psPAX2 and pMD2.G using Jetprime transfection reagent. To establish stable cell lines, GBM cells were transduced with the harvested lentivirus with 8 µg/mL polybrene. After 72 h of transduction, cells were selected with 0.5 µg/mL puromycin for one week and expanded as polyclonal populations.

### 2.2. Xenograft Tumor Model

All experiments involving nude mice with intracranial tumors were conducted in accordance with the National Institutes of Health Guidelines for the Care and Use of Laboratory Animals and were approved by the Animal Research Committee of Nanchang University. Sample size was determined based on previous research [26]. Five-week-old male BALB/c nude mice (*n* = 16) were assigned to groups by simple randomization using a random-number generator (*n* = 8 per group). Under anesthesia, 5 × 10^5^ luciferase-labeled LN229 control cells or LN229 shSELENOS cells, suspended in 10 µL of Hank’s Balanced Salt Solution (HBSS), were stereotactically injected into the right frontal lobe (2.5 mm lateral and 1 mm anterior to the bregma, 3.0 mm deep from the cortical surface) using a stereotaxic apparatus. Tumor progression was monitored via IVIS bioluminescence imaging at weeks 1, 2, 3, and 4 after implantation, following intraperitoneal injection of luciferin substrate. Photon flux values at each time point were used to assess tumor burden. Predefined exclusion criteria included animals that died within 48 h after intracranial inoculation (indicative of surgical failure). Humane endpoints (≥20% weight loss or severe clinical deterioration) were used for ethical termination and not for exclusion from analysis. No animals met the predefined exclusion criteria. Group comparisons were performed using two-sided Student’s *t*-test. Data are means ± SEMs. This study was reported in accordance with the ARRIVE criteria [27].

### 2.3. Data Collection

The RNA-seq data from TCGA and GTEx datasets processed uniformly through the Toil pipeline were downloaded from UCSC Xena (https://xenabrowser.net/datapages/, accessed on 1 August 2025). The gene expression was transformed into log2(TPM+1). ssGSEA analysis of selenoproteins was conducted using the GSVA package (v2.2.0) with the ssGSEA method. The CPTAC-GBM dataset was used for evaluating selenoprotein expression between normal and GBM tissues. Public glioma datasets from GlioVis (http://gliovis.bioinfo.cnio.es, accessed on 26 April 2026) were used to analyze survival outcomes. Survival analysis was conducted using the log-rank test.

### 2.4. Single-Cell RNA Sequencing Reanalysis

The core GBmap dataset (including raw and normalized counts, integrated embeddings, cell-type annotations, and technical and clinical metadata) was downloaded via cellxgene (https://cellxgene.cziscience.com/collections/999f2a15-3d7e-440b-96ae-2c806799c08c, accessed on 1 September 2025). This dataset comprises IDH-wildtype glioma scRNA-seq profiles collected from 16 independent studies spanning diverse sequencing platforms and sample preparation protocols, encompassing over 330,000 cells from 109 patients [25]. Then, we selected samples with annotated regional information, including 10,591 cells from the tumor core and 7846 cells from the tumor periphery. We analyzed selenoprotein expression across various cell types using the Seurat package (v4.3.0). Dimensionality reduction and clustering were performed using Seurat (v4.3.0) in R (v4.4.1).

### 2.5. Acquisition of Selenoproteins and Scoring

The human genome contains 25 genes encoding selenocysteine-containing proteins (selenoproteins) [28]. To investigate the role of selenoproteins in glioma at single-cell resolution, we applied five scoring methods (AUCell, UCell, singscore, ssGSEA, and AddModuleScore) [29,30,31,32] to compute selenoprotein activity scores for each cell. Scores from each method were first Z-standardized and then min–max normalized to ensure comparability. To integrate complementary rank-based and expression-based scoring strategies, a composite selenoprotein score (Scoring) was calculated by summing the five normalized values with equal weighting. Given the lack of prior evidence favoring any single method, equal weighting was applied to avoid bias toward a specific scoring strategy. Malignant cells were subsequently stratified into SehighMali and SelowMali groups based on the median Scoring value within malignant cells.

### 2.6. Machine Learning Model Construction

The Mime1 R package (v0.0.0.9000) [33] was used to evaluate whether selenoprotein gene expression could predict glioma prognosis. Prognosis-associated selenoprotein genes were first identified by univariate Cox regression (unicox.filter.for.candi = TRUE, unicox_p_cutoff = 0.05). These genes were then used to train prognostic models across all 101 predefined combinations of ten algorithms, using the TCGA cohort as the training dataset. Model performance was assessed in the CGGA and Rembrandt cohorts using Harrell’s concordance index (C-index), and the model with the highest average C-index across datasets was selected. Parameter settings (mode = “all”, nodesize = 5, seed = 5,201,314) followed the recommended workflow to ensure reproducibility.

### 2.7. Functional Enrichment Analysis

To understand the functional characteristics of the different cell subpopulations, we first identified highly expressed genes for each subpopulation using the FindAllMarkers function (adjusted *p* < 0.05, log2FC  >  0.25). Functional enrichment analysis of the marker genes for each subpopulation was then performed using the GO databases through the R package clusterProfiler (v4.16.0) [34]. An FDR of less than 0.05 was considered significant. Functional enrichment of each cell type was analyzed by scoring hallmark gene sets using the irGSEA package (v3.3.3). For bulk RNA-seq data, GSVA (v2.2.0) [35] was used to assign functional scores to samples based on predefined gene sets.

### 2.8. Cell Communication Analysis

Cellular communication between SehighMali and other cells was initially analyzed using CellChat (v2.2.0) [36]. First, the overall communication strength and frequency between all SehighMali cells and myeloid subpopulations were calculated. Key cell types interacting with SehighMali were identified. To infer specific signaling interactions between SehighMali cells and other cells and assess their impact on downstream target genes, we used the R package NicheNet (v2.2.0) [37]. Here, SehighMali served as signal senders, and myeloid cells as receivers. The top 30 ligands, receptors, and target genes ranked by aupr_corrected were visualized in a heatmap.

### 2.9. Bulk Deconvolution Analysis

To assess the infiltration of different cell subpopulations in the TCGA dataset, we estimated cell abundance in each sample using CIBERSORTx (https://cibersortx.stanford.edu/, accessed on 1 Octorber 2025) [38]. To obtain a balanced and computationally manageable single-cell–derived signature matrix, we used a two-step procedure. First, to prevent highly abundant populations from dominating the signature and to meet the input size constraints of CIBERSORTx, we randomly sampled 500 cells per cell type. The resulting signature matrix, together with the TCGA bulk expression mixture file, was then submitted to CIBERSORTx for deconvolution. Deconvolution analysis was also performed in the CGGA and Rembrandt validation cohorts, where samples were stratified into high and low SehighMali groups based on the median score, followed by survival analysis.

### 2.10. Spatial Transcriptomics Analysis

Spatial transcriptomic analysis was performed using two independent GBM tissue sections (#UKF269_T and #UKF304_T) retrieved from publicly available datasets (https://datadryad.org/dataset/doi:10.5061/dryad.h70rxwdmj, accessed on 1 June 2025) [23]. Tissue segmentation and spatial domain annotation, including identification of tumor core, transitional zone, and infiltrative cortex, were conducted using the SPATA2 package (v3.1.4) [39]. To assess regional selenoprotein expression, we calculated module scores for curated selenoprotein gene sets using the AddModuleScore function in Seurat, and visualized their spatial distribution across tissue sections. Copy number variations (CNVs) were inferred from spatial transcriptomic data using the inferCNV package (v1.24.0) to evaluate genomic alterations potentially associated with elevated selenoprotein expression. To estimate immune cell infiltration, SpaCET (v1.3.0) [40] was applied to deconvolute spatial transcriptomic profiles and quantify immune lineage abundance in each spatial spot. The spatial relationship between SehighMali regions and immune populations was further explored by calculating transcriptomic distances and visualizing colocalization patterns. Finally, cell–cell communication analysis was conducted using the COMMOT framework [41] to identify spatially enriched ligand–receptor interactions. In particular, CSF1–CSF1R signaling activity was examined within SehighMali regions to assess potential crosstalk between tumor and immune compartments.

### 2.11. Transwell Migration and Invasion Assay

Cell invasion was assessed using Matrigel-coated Transwell inserts (8-μm pores, Millipore, Billerica, MA, USA). Stably transfected 2 × 10^5^ glioma cells (serum-free DMEM) were seeded in the upper chamber, while the lower chamber contained DMEM with 10% FBS as a chemoattractant. After 22 h, non-invading cells were removed, and invaded cells were methanol-fixed, PBS-washed, and stained with 0.1% crystal violet. Cells were counted in five random fields per insert across three independent experiments, and images were captured.

### 2.12. Quantitative Real-Time PCR

Total RNA was extracted using TRIzol reagent (Invitrogen, Waltham, MA, USA) and reverse-transcribed with the iScript™ Reverse Transcription Supermix (Bio-Rad, Hercules, CA, USA) following the manufacturer’s instructions. Quantitative PCR was performed using SYBR Green Master Mix (Thermo Fisher Scientific, Waltham, MA, USA). Primer sequences for all genes are provided in Table S1.

### 2.13. Colony Formation Assays

LN229 or U87MG cells were seeded in a 6-well culture (800 cells per well) and cultured for about 10 or 12 days. Cells were fixed with 4% paraformaldehyde and stained with dye of 0.1% crystal violet. The number of anchorage-dependent colonies (defined as clusters of >50 cells) was measured by inverted microscope. Cell colonies were observed, photographed, and counted.

### 2.14. Enzyme-Linked Immunosorbent Assay (ELISA)

The concentration of CSF1 in supernatants was measured with a CSF1 enzyme-linked immunosorbent assay kit (Sino Biological, Beijing, China), according to the manufacturer’s protocol. Briefly, 100 μL of cultured cell medium was added to each well, the plate was covered and incubated for 2 h at room temperature. After washing, wells were incubated with the secondary antibody for 1 h at room temperature. The liquid was then removed, and wells were washed three times. Substrate solution was added, and the plate was incubated for 20 min at room temperature, protected from light. The reaction was stopped by adding 100 μL of stop solution to each well, followed by gentle mixing. The absorbance was measured at 450 nm within 10 min of adding the stop solution.

### 2.15. IHC

Tissue sections from GBM xenografts were prepared for immunohistochemical staining by deparaffinization and rehydration with an ethanol series. Antigen retrieval was then performed using sodium citrate or tris-EDTA buffer according to the antibody manufacturer’s instructions. Sections were immersed in 3% H_2_O_2_ solution in phosphate-buffered saline at room temperature for 15 min to block endogenous peroxidases and were then blocked with 3% bovine serum albumin in tris-buffered saline solution at room temperature for 60 min. The sections were incubated overnight with primary antibody at 4 °C, followed by immunohistochemical staining using horseradish peroxidase-conjugated secondary antibodies and diaminobenzidine as the chromogen.

### 2.16. CCK8

Cell proliferation ability was determined by CCK-8 assay, with each experimental condition replicated five times. Glioma cells were seeded and cultured in a 96-well microplate at a density of 3000 cells/well. After the addition of 10 μL of CCK-8 reagent in each well, glioma cells were incubated at 37 °C for 1 h. The absorbance was analyzed at 450 nm at 0, 24, 72 and 120 h using a microplate reader.

### 2.17. Therapeutic Response Analysis and Molecular Docking

Drug sensitivity for each TCGA patient was predicted using OncoPredict version 1.2 [42], based on pharmacogenomic profiles from the Cancer Therapeutics Response Portal (CTRP v2.0). Lower scores indicate higher predicted sensitivity. To validate findings at the single-cell level, Beyondcell [43] was applied to scRNA-seq data to compare drug responses between SehighMali and SelowMali subpopulations. Molecular docking between SELENOS and candidate compounds was performed using CB-Dock2 [44], a blind docking framework integrating cavity detection, docking, and homology-based modeling.

### 2.18. Statistical Analysis

Statistical analyses and visualizations were performed in R (v4.3.0). Differences between groups were compared using the Wilcoxon test. Survival analyses were conducted using the Kaplan–Meier method, with statistical significance determined by the log-rank test. Cox regression assessed correlations between variables and patient overall survival (OS), using the median or optimal cut-off as thresholds. Statistical significance was set at *p*  <  0.05. Correlation analyses, given the non-normal data distribution, were conducted using the Spearman method.

## 3. Results

### 3.1. Expression Landscape and Prognostic Significance of Selenoproteins in Gliomas

**Figure 1:**
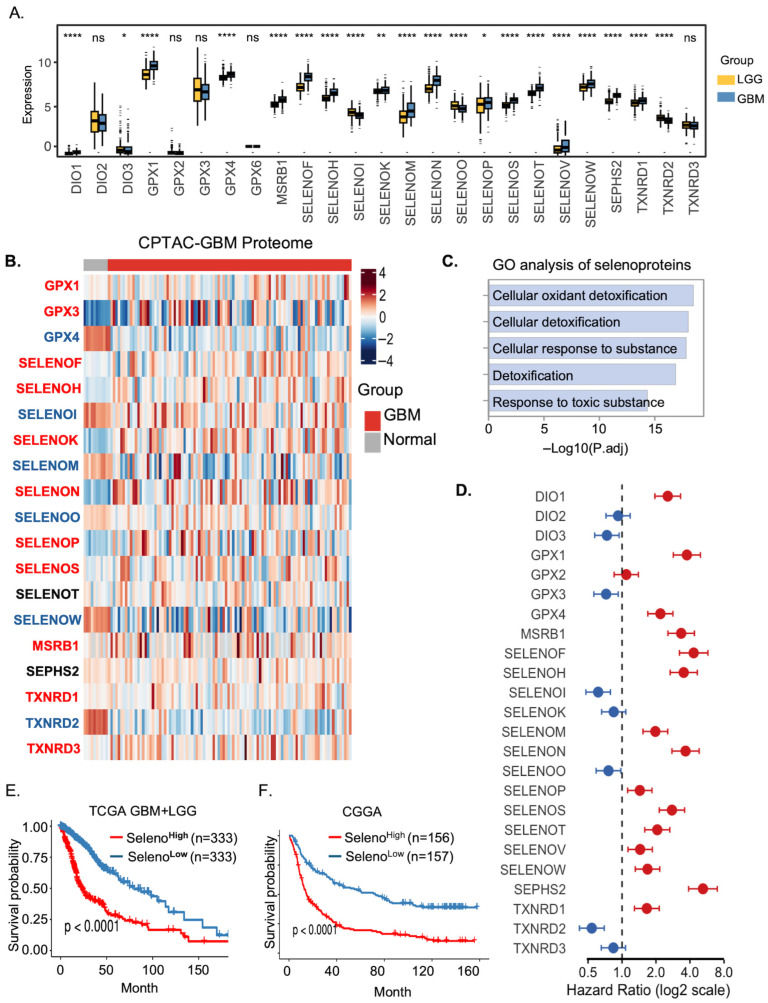
Expression profiling and prognostic relevance of selenoprotein-related genes in gliomas. (**A**) Box plots showing expression levels of selenoproteins in lower-grade glioma (LGG, yellow) and glioblastoma (GBM, blue) samples based on TCGA RNA-seq data. * *p* < 0.05; ** *p* < 0.01; **** *p* < 0.0001; ns, not significant. (**B**) Heatmap of protein expression levels of selenoprotein-related genes in GBM (red) and normal brain tissues (grey) from the CPTAC-GBM proteomics dataset. Protein abundance is scaled by Z-score. Proteins significantly upregulated in GBM tissues (*p* < 0.05) are labeled in red, those significantly downregulated (*p* < 0.05) are labeled in blue, and proteins without significant differences are shown in black. (**C**) Representative enriched GO pathways for the selenoproteins. (**D**) Forest plot showing hazard ratios (log2 scale) for selenoprotein genes in glioma from univariate Cox regression analysis. (**E**,**F**) Kaplan–Meier survival curves of glioma patients stratified by high (red) and low (blue) selenoprotein gene-set scores (median split) in the TCGA and CGGA cohorts (log-rank test, *p* < 0.0001).

Previous studies have indicated that various selenoproteins are involved in glioma progression. To further investigate their roles, we integrated transcriptomic data from lower-grade glioma (LGG) and GBM patients in the TCGA dataset and analyzed the differential expression of selenoprotein-related genes. Most of the selenoproteins exhibited significant expression differences between LGG and GBM. Notably, the majority of selenoproteins were upregulated in GBM, except for SELENOI, SELENOO, TXNRD2, TXNRD3, GPX2, GPX3, GPX6, DIO2 and DIO3 (Figure 1A). To validate these findings at the protein level, we further analyzed publicly available proteomic data comparing GBM and normal brain tissues. Consistently, most selenoproteins were also found to be upregulated in GBM at the proteomic level, reinforcing their potential oncogenic roles in glioma (Figure 1B). To gain insights into the potential biological functions of selenoproteins in glioma, we first performed functional enrichment analysis of the 25 selenoprotein genes. The results revealed that these genes are primarily involved in detoxification and cellular responses to toxic substances (Figure 1C). We then performed univariate Cox regression analysis to evaluate their prognostic significance. Based on median expression levels, 15 selenoproteins—including DIO1, GPX1, GPX4, MSRB1, SELENOF, SELENOH, SELENOM, SELENON, SELENOP, SELENOS, SELENOT, SELENOV, SELENOW, SEPHS2, and TXNRD1 were significantly associated with poor prognosis, while four genes (DIO3, GPX3, SELENOI, and TXNRD2) were identified as protective factors (Figure 1D). Furthermore, ssGSEA analysis showed that higher selenoprotein scores were significantly correlated with worse survival outcomes in both TCGA and CGGA cohorts (Figure 1E,F), highlighting their functional relevance in glioma progression.

### 3.2. Machine Learning-Based Prognostic Modeling Using Selenoprotein Gene Expression

**Figure 2:**
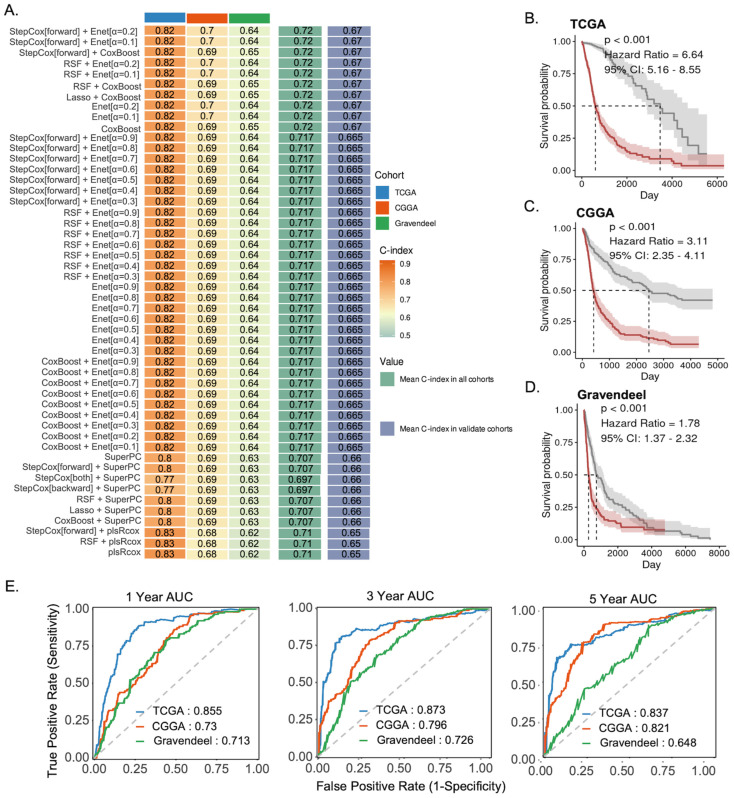
Prognostic modeling based on selenoprotein-related genes in glioma. (**A**) Concordance index (C-index) of machine learning models trained on selenoprotein gene–expression features across the TCGA, CGGA, and Gravendeel cohorts. (**B**–**D**) Kaplan–Meier survival curves comparing high- and low-risk groups predicted by the optimal selenoprotein-based model in the TCGA (**B**), CGGA (**C**), and Gravendeel (**D**) datasets. (**E**) Time-dependent ROC curves evaluating the model’s ability to predict 1–, 3–, and 5–year survival across the three cohorts.

We subsequently constructed a glioma prognostic model based on selenoprotein gene expression across three transcriptomic datasets, including one training cohort and two independent validation cohorts. Using the Mime platform, which integrates 10 machine learning algorithms, a total of 117 models were developed. Among them, we found that StepCox[forward] + Enet[α = 0.2] achieved the highest concordance index (C-index) across all validation sets, demonstrating robust predictive performance (Figure 2A). Based on the median risk score calculated from the StepCox[forward] + Enet[α = 0.2] model, glioma patients were stratified into high- and low-risk groups. In all cohorts, patients in the high-risk group exhibited significantly worse survival outcomes (Figure 2B–D). The model also demonstrated strong predictive accuracy, with high area under the curve (AUC) values for 1–, 3–, and 5–year survival (Figure 2E). Compared to other models, it consistently achieved superior C-index performance across nearly all datasets (Figure S1). To further assess the clinical applicability of the selenoprotein-based risk score, we evaluated its performance in both the TCGA and CGGA cohorts. After adjustment for key clinical and molecular variables, the risk score remained an independent prognostic factor in multivariate Cox analyses (Figure S2A,C). It also demonstrated superior predictive performance, showing consistently higher C-index values than conventional clinical markers in both datasets, and the combined model incorporating both the risk score and clinical variables achieved the highest predictive accuracy (Figure S2B,D). Under the WHO 2021 glioma molecular classification, Kaplan–Meier survival analyses were performed independently within each subtype, and *p* values were adjusted for multiple comparisons using the Benjamini–Hochberg method. After correction, the model remained significantly associated with survival in oligodendrogliomas (FDR = 0.0030), while a trend toward significance was observed in IDH-mutant astrocytomas (unadjusted *p* < 0.05, FDR = 0.052). In the IDH-wildtype glioblastoma subgroup, the high-risk group showed a consistent trend toward poorer survival, although the difference was not statistically significant (*p* = 0.124; Figure S2E). These results indicate that the prognostic model exhibits subtype-dependent performance, with potential clinical relevance in specific molecular contexts.

### 3.3. Single-Cell Transcriptomics Identifies a Selenoprotein-Enriched Malignant Subpopulation (SehighMali)

**Figure 3:**
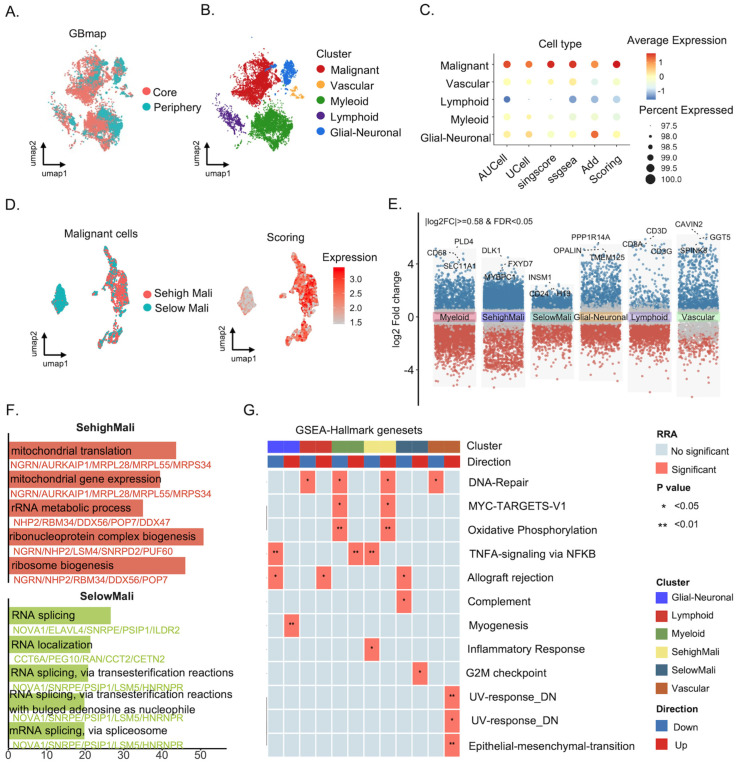
Single-cell transcriptomic characterization of selenoprotein-associated programs in glioblastoma. (**A**) UMAP visualization of cells from GBM samples (GBmap), annotated by spatial origin (tumor core vs. periphery). (**B**) Cell type annotation based on transcriptomic clustering, including malignant, vascular, myeloid, lymphoid, and glial-neuronal populations. (**C**) Dot plot displaying the expression of selenoprotein across cell types. Dot size represents the percentage of cells expressing the signature, and dot color reflects the averaged expression level. (**D**) UMAP visualization of malignant cells stratified into SehighMali and SelowMali groups based on selenoprotein Scoring. (**E**) Volcano plots illustrating differentially expressed genes between major cell clusters and malignant subsets, with key markers labeled. (**F**) Gene ontology enrichment analysis of SehighMali and SelowMali cells reveals distinct biological programs. (**G**) Heatmap showing the distribution of significantly enriched Hallmark gene sets across cell clusters, identified by the robust rank aggregation (RRA) algorithm. Asterisks in each grid cell indicate the statistical significance (*p*-value). Direction in the legend denotes whether the enrichment of each gene set in a given cluster is higher (Up) or lower (Down) compared to other clusters.

To investigate the role of selenoproteins in glioma at a higher resolution, we applied five single-cell scoring methods to calculate selenoprotein expression scores and then computed their average, referred to as “Scoring.” To assess the reliability of the composite scoring framework, we performed correlation analysis between Scoring and each individual method (AUCell, UCell, singscore, ssGSEA, and AddModuleScore). All methods showed strong concordance with Scoring (Spearman r > 0.8, FDR < 0.001; Figure S3A), indicating consistent biological patterns across different approaches. Across major cell lineages, malignant cells exhibited the highest selenoprotein scores, followed by glial-neuronal cells (Figure 3A–C). Selenoprotein scores were significantly higher in core-derived malignant cells compared to peripheral malignant cells (Figure S3B), highlighting intra-tumoral spatial heterogeneity. Malignant cells were stratified into two groups (SehighMali and SelowMali) based on the median Scoring value (Figure 3D). Pairwise consistency analysis further demonstrated high agreement in classification across methods (81.6–87.0%; Table S2), supporting the robustness of Scoring as a representative metric. Transcriptomic profiling revealed distinct gene expression patterns between these two groups. For instance, DLK1 and MYBPC1 were highly expressed in the SehighMali group, whereas genes such as CD24 and INSM1 were upregulated in SelowMali cells (Figure 3E). Notably, GO enrichment analysis showed that SehighMali-specific genes were associated with mitochondrial translation and ribosome biogenesis, suggesting increased demands for energy metabolism and translational activity (Figure 3F). In contrast, SelowMali cells exhibited enrichment in RNA splicing pathways. GSEA further indicated that the SehighMali cells displayed enhanced DNA repair, upregulation of MYC targets, and increased oxidative phosphorylation, along with reduced inflammatory responses (Figure 3G). Notably, unsupervised clustering of malignant cells revealed pronounced, non-random enrichment patterns: clusters 1, 5, and 10 were enriched for high-scoring cells, whereas clusters 0 and 9 were largely composed of low-scoring cells (Figure S3C,D). These patterns indicate that SehighMali represents a coherent transcriptional program rather than an artefact of score dichotomization. Comparison with established malignant cell states (OPC-like, NPC-like, MES-like, AC-like) further showed that SehighMali does not collapse onto any single known state (Figure S3F). Notably, pseudotime analysis indicated a continuous trajectory, with SelowMali cells enriched at early stages and SehighMali cells at later stages, supporting a dynamic rather than static state model (Figure S3G).

### 3.4. SehighMali Cells Exhibit Elevated Outgoing Signaling and Interact Preferentially with Myeloid Populations

**Figure 4:**
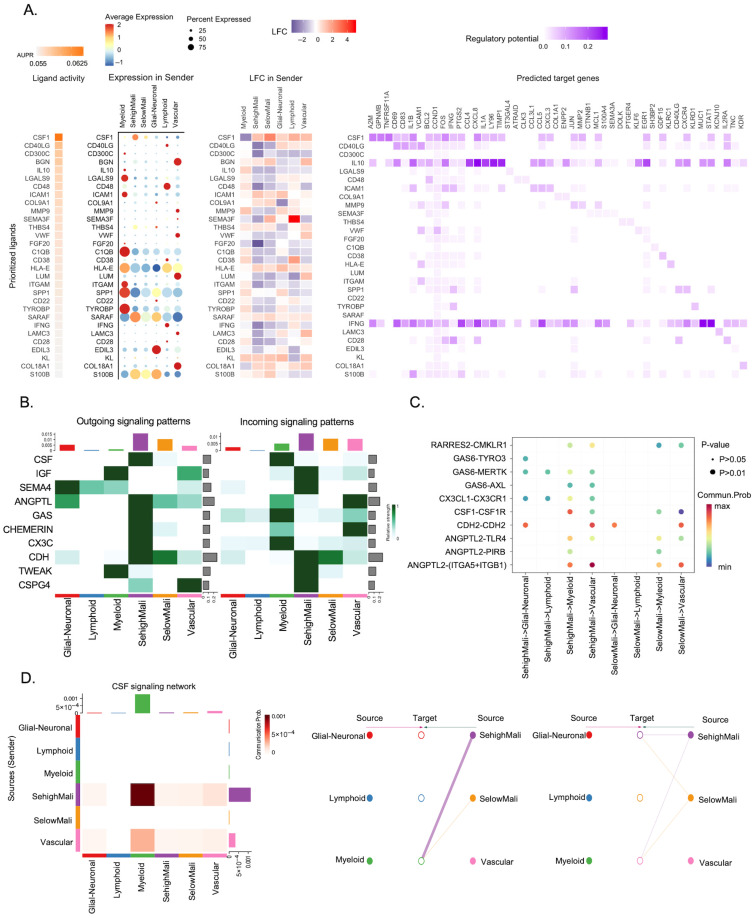
Cell–cell communication analysis reveals dominant signaling features of SehighMali cells. (**A**) NicheNet analysis identifying top ligand–target signaling axis mediated by SehighMali cells. Left panel shows ligand activity scores (AUPR) based on their ability to predict downstream gene expression in receivers. Dot plots depict average expression, percent expression, and log-fold change (LFC) of ligands across sender populations. Right panel shows predicted ligand–target gene associations with regulatory potential. (**B**) Outgoing (**left**) and incoming (**right**) signaling strength of major pathways across cell populations, inferred using CellChat. (**C**) Dot plot comparing outgoing ligand–receptor interactions between SehighMali and SelowMali across major cell populations. (**D**) Detailed CSF signaling network showing dominant information flow from SehighMali cells to myeloid populations. Left panel shows quantitative interaction strengths; right panels visualize directional source–target relationships.

To further examine the functional relevance of ligand expression in SehighMali cells, we used NicheNet to rank ligands by expression and predicted regulatory potential. CSF1 emerged as the highest-scoring ligand in SehighMali cells, showing both strong expression and the greatest influence on downstream target genes. A corresponding heatmap supported the central role of CSF1-mediated signaling in pathways related to immune regulation (Figure 4A). We next characterized the broader communication landscape using CellChat. SehighMali cells showed the strongest outgoing signaling across multiple pathways (Figure 4B). Analysis of ligand–receptor interactions further revealed that CSF1–CSF1R, together with GAS6–AXL and CX3CL1–CX3CR1, were preferentially enriched between SehighMali cells and myeloid populations (Figure 4B,C). Examination of the CSF signaling network indicated that SehighMali cells were the predominant source of CSF1, with signals directed mainly toward myeloid cells (Figure 4D). These findings support a model in which SehighMali cells foster an immunosuppressive microenvironment through CSF1-driven signaling, thereby promoting glioma progression.

### 3.5. Spatial Mapping of Selenoproteins and Immune Interactions

**Figure 5:**
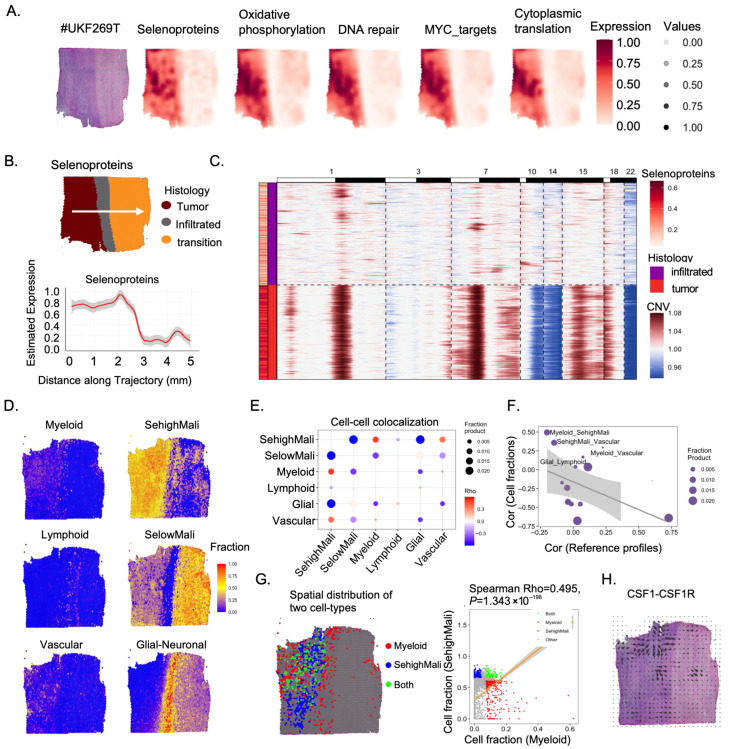
Spatial transcriptomics reveals tumor core localization and immune interaction of SehighMali cells. (**A**) Spatial expression patterns in a representative GBM sample (UKF#269T), showing selenoprotein gene signature and associated pathways including oxidative phosphorylation, DNA repair, MYC targets, and cytoplasmic translation. (**B**) Top: histological segmentation of tumor, infiltrated, and transition zones along a defined trajectory. Bottom: smoothed expression of selenoprotein genes shows peak signal localized within the tumor core. (**C**) Spatial heatmap of gene expression (rows) across spatial barcodes (columns) ordered along histological and inferred CNV gradient. Selenoprotein signature genes are enriched in regions with tumor-like CNV profiles. (**D**) Spatial mapping of key cell types inferred from deconvolution, highlighting SehighMali and SelowMali malignant subgroups, as well as myeloid, lymphoid, vascular, and glial-neuronal compartments. (**E**) Cell–cell colocalization matrix showing spatial proximity patterns across inferred cell types, with strong enrichment between SehighMali and myeloid cells. (**F**) Right: correlation between cell-type colocalization and transcriptomic reference overlap. (**G**) Left: spatial distribution of SehighMali (red) and myeloid (blue) cells shows co-localization within tumor core regions. Right: correlation of SehighMali and myeloid cell fractions across spatial bins. (**H**) Spatial distribution of CSF1-CSF1R signaling within the GBM sample by COMMOT algorithym.

To further validate the above findings at the spatial transcriptomic level, we first examined pathway activities associated with selenoprotein expression across a GBM tissue section (sample #UKF269T). Spatial regions with high selenoprotein scores exhibited increased activity in pathways related to oxidative phosphorylation, DNA repair, MYC targets, and cytoplasmic translation, indicative of a metabolically active and proliferative malignant cell state (Figure 5A). We next applied the SPATA2 package to segment this tissue section into three anatomical regions: the tumor core, transitional zone, and infiltrative cortex (Figure 5B). Across this spatial continuum, selenoprotein expression progressively decreased from the tumor core toward the infiltrative cortex. Consistently, inferCNV analysis revealed a significant positive correlation between selenoprotein expression and copy number variation, suggesting that genomic alterations may contribute to elevated selenoprotein levels in malignant regions (Figure 5C). To further assess the spatial association between SehighMali and immune infiltration, we performed spatial transcriptomic analysis on two independent GBM samples. Using the SpaCET deconvolution algorithm, we estimated immune cell abundances within each spatial spot (Figure 5D and Figure S4A). All samples demonstrated substantial infiltration of both SehighMali and myeloid cells, with consistent spatial colocalization (Figure 5E–G and Figure S4B–D). Importantly, the transcriptomic similarity between SehighMali and myeloid cells was relatively low, indicating that their colocalization is not due to shared expression profiles but likely reflects true physical proximity within the tumor microenvironment (Figure 5F and Figure S4C). Finally, cell–cell communication analysis using COMMOT revealed enrichment of CSF1–CSF1R signaling within the SehighMali regions, further implicating paracrine interactions between malignant and myeloid cells (Figure 5H and Figure S4E). Further analysis revealed that in myeloid cells, the M2 marker CD163 exhibited higher expression and broader distribution compared with the M1 macrophage marker CD86, and showed more extensive co-localization with SehighMali cells, suggesting its association with an immunosuppressive microenvironment (Figure S4F,G).

### 3.6. SehighMali Abundance Associates with Immunosuppressive Signatures and Adverse Clinical Features

**Figure 6:**
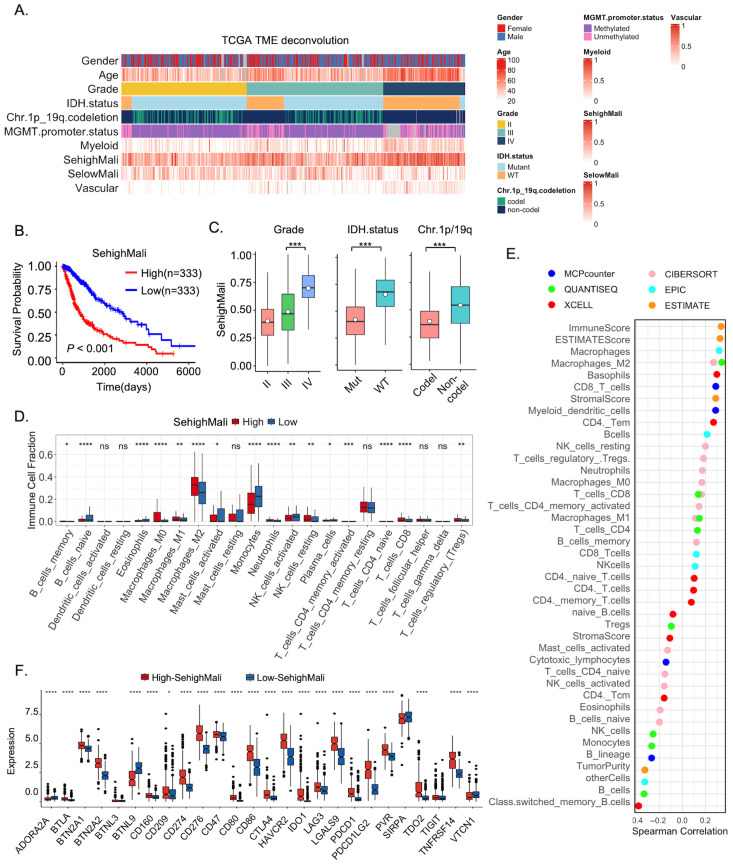
Clinical and immunological associations of SehighMali cells in bulk glioma datasets. (**A**) Heatmap of TCGA glioma samples showing deconvolved cell-type fractions (SehighMali, SelowMali, myeloid, vascular) and clinical variables including gender, age, WHO grade, IDH mutation, 1p/19q codeletion, and MGMT promoter methylation status. (**B**) Kaplan–Meier survival curve comparing overall survival between patients with high and low SehighMali abundance in TCGA cohort. Groups stratified by median value, *p* < 0.001 (log-rank test). (**C**) SehighMali abundance across tumor grade, IDH status, and 1p/19q codeletion status. SehighMali is significantly enriched in IDH-wildtype, Grade IV, and non-codeleted gliomas. *** *p* < 0.001. (**D**) Comparison of immune cell infiltration between high- and low-SehighMali groups based on CIBERSORT deconvolution. * *p* < 0.05, ** *p* < 0.01, *** *p* < 0.001, **** *p* < 0.0001; ns, not significant. (**E**) Correlation of SehighMali abundance with immune microenvironment features across six algorithms (MCP-counter, xCell, EPIC, CIBERSORT, QUANTISEQ, ESTIMATE). (**F**) Expression levels of immune checkpoint genes in high- and low-SehighMali groups. (* *p* < 0.05, **** *p* < 0.0001).

To further investigate the prognostic relevance of the SehighMali subpopulation in a larger cohort, we used CIBERSORTx to quantify the abundance of SehighMali and selowMali in the TCGA-GBMLGG dataset (Figure 6A). Univariate Cox regression analysis revealed that a higher abundance of SehighMali was significantly associated with poorer overall survival in three independent cohorts (Figure 6B and Figure S5A,B). Moreover, the abundance of SehighMali cells was negatively correlated with key clinical and molecular features of malignant gliomas (Figure 6C). Given the complex and multifaceted roles of immune infiltration in glioma, we next examined the relationship between SehighMali and the tumor immune microenvironment. We applied the CIBERSORT algorithm to estimate the relative proportions of 22 immune cell types. Compared to the low SehighMali group, the high SehighMali group showed significantly elevated proportions of regulatory T cells (Tregs) and M2 macrophages, whereas the low SehighMali group exhibited enriched monocyte and activated NK cell infiltration (*p* < 0.01; Figure 6D). Subsequently, we integrated five additional immune deconvolution algorithms to validate the immune landscape. Despite variability across methods, SehighMali consistently showed a positive correlation with macrophage infiltration. In addition, SehighMali was positively associated with both the immune score and ESTIMATE score but negatively associated with tumor purity (Figure 6E). Moreover, considering the significant impact of immune checkpoint molecules on tumor immunotherapy, we analyzed the expression levels of immune checkpoint genes between the high- and low-SehighMali subgroups. We found that the expression levels of inhibitory checkpoint genes, including HAVCR2, PDCD1, CD274 were elevated in high-SehighMali tumors (Figure 6F), suggesting an immunosuppressive phenotype. In contrast, patients with high SelowMali exhibited better overall survival, lower expression of immune-checkpoint genes, and immune features broadly opposite to those of SehighMali, including stronger associations with immune activation (Figure S6A–E).

### 3.7. SELENOS Links the SehighMali Program to Glioma Malignancy and Myeloid Immunosuppression

**Figure 7:**
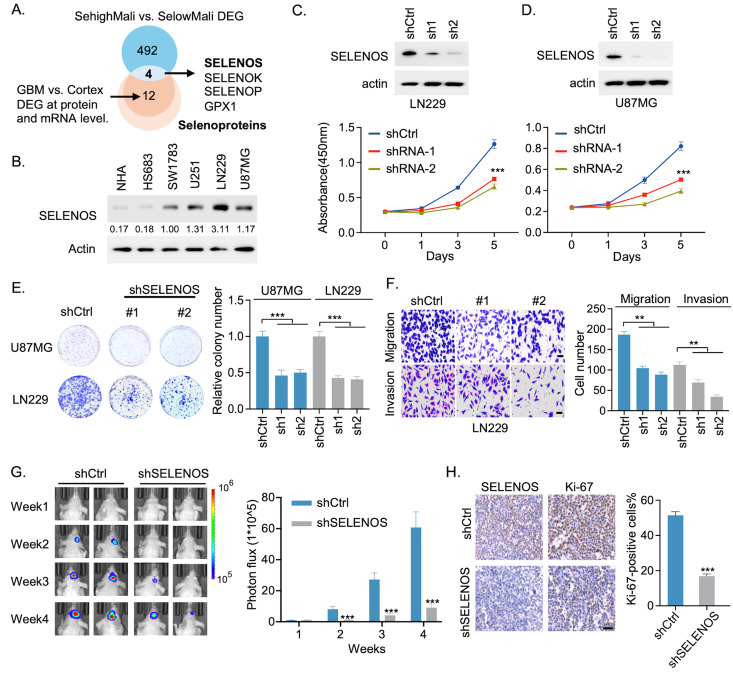
Knockdown of SELENOS suppresses glioma cell proliferation, migration, and invasion in vitro and in vivo. (**A**) Venn diagram showing overlap between differentially expressed genes in SehighMali vs. SelowMali cells (logFC > 0.25, FDR < 0.05) and GBM vs. normal cortex at both RNA and protein levels. (**B**) Western blot analysis of SELENOS expression across glioma cell lines and normal human astrocytes (NHA). (**C**,**D**) Western blot confirmation of SELENOS knockdown using two independent shRNAs in LN229 (**C**) and U87MG (**D**) cells, with corresponding cell proliferation curves. *** *p* < 0.001. (**E**) Colony formation assays showing impaired clonogenicity in SELENOS-depleted U87MG and LN229 cells. Quantification of colony numbers is shown at right. *** *p* < 0.001. (**F**) Migration and invasion assays in LN229 cells following SELENOS knockdown. ** *p* < 0.01. Scale bar, 10 μm. (**G**) Bioluminescent imaging of xenografts from luciferase-labeled LN229 cells with or without SELENOS knockdown. Photon flux was quantified (right panel). *n* = 8. *** *p* < 0.001. (**H**) Sections from tumor xenografts derived from LN229 cells were immunostained using SELENOS and Ki-67. Scale bar, 50 μm. *** *p* < 0.001. All bar plot data are the means ± SEMs.

**Figure 8:**
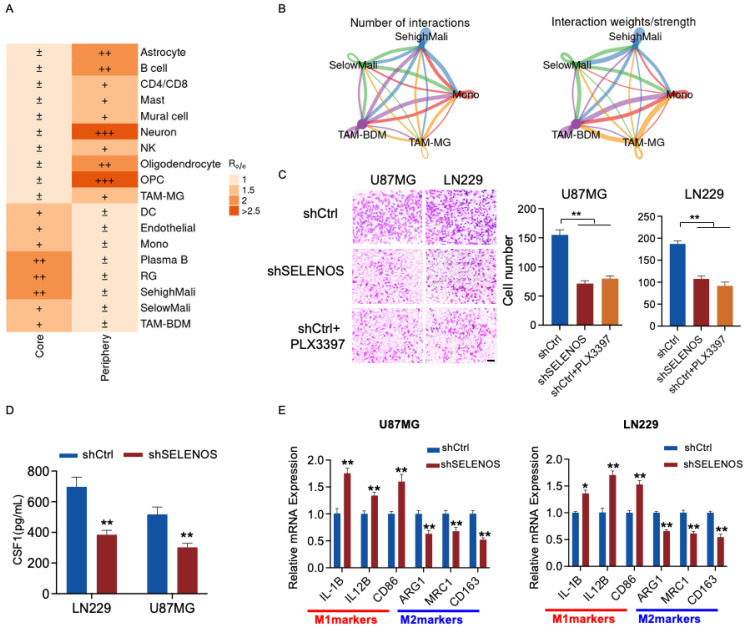
SELENOS influences CSF1 expression and TAM-related responses in glioma. (**A**) Tissue-enrichment patterns of major cell types shown by Ro/e scores, indicating their relative preference for core versus peripheral tumor regions. (**B**) Network diagram illustrating the intercellular communication strength between SehighMali, SelowMali, and distinct myeloid populations. (**C**) Transwell migration assays of THP-1–derived macrophages treated with conditioned media from shCtrl or shSELENOS glioma cells. PLX3397 (1 μM) was applied to block CSF1R signaling. Scale bar = 50 μm. ** *p* < 0.01. (**D**) CSF1 levels in conditioned media collected from U87MG and LN229 cells transfected with shCtrl or shSELENOS, measured by ELISA. ** *p* < 0.01. (**E**) qRT-PCR analysis of M1- and M2-associated markers in THP-1–derived macrophages stimulated with conditioned media from the indicated glioma cells. * *p* < 0.05, ** *p* < 0.01.

To identify potential effectors of the SehighMali transcriptional program, we analyzed the differentially expressed genes between SehighMali and SelowMali cells. Among these genes, four selenoproteins (SELENOS, SELENOK, SELENOP and GPX1) were consistently upregulated in GBM compared to normal cortex at both the transcriptomic and proteomic levels (Figure 7A). Notably, SELENOS has been relatively underexplored in glioma, despite its reported prognostic value in lower-grade glioma [15]. We therefore investigated the role of SELENOS in glioma malignancy. Western blotting confirmed that SELENOS expression was elevated in GBM cell lines compared to the lower-grade glioma cell line SW1783 and normal human astrocytes (NHA) (Figure 7B and Figure S7). Knockdown of SELENOS using shRNA in LN229 and U87MG cells significantly reduced cell proliferation, colony formation, and migration in vitro (Figure 7C–F and Figure S7). In orthotopic xenograft models, SELENOS depletion suppressed tumor growth and reduced Ki-67 expression (Figure 7G,H), supporting a functional role for SELENOS in glioma progression. Importantly, CellChat analysis showed that SehighMali cells engaged more strongly with TAM-BDM than with TAM-MG populations. In functional assays, SELENOS knockdown reduced TAM recruitment, an effect comparable to CSF1R inhibition (PLX3397). SELENOS depletion reduced CSF1 levels of glioma and shifted macrophages toward a more pro-inflammatory phenotype (Figure 8A).

### 3.8. SehighMali Cells Are Resistant to Temozolomide but May Be Targetable by Alternative Compounds

**Figure 9:**
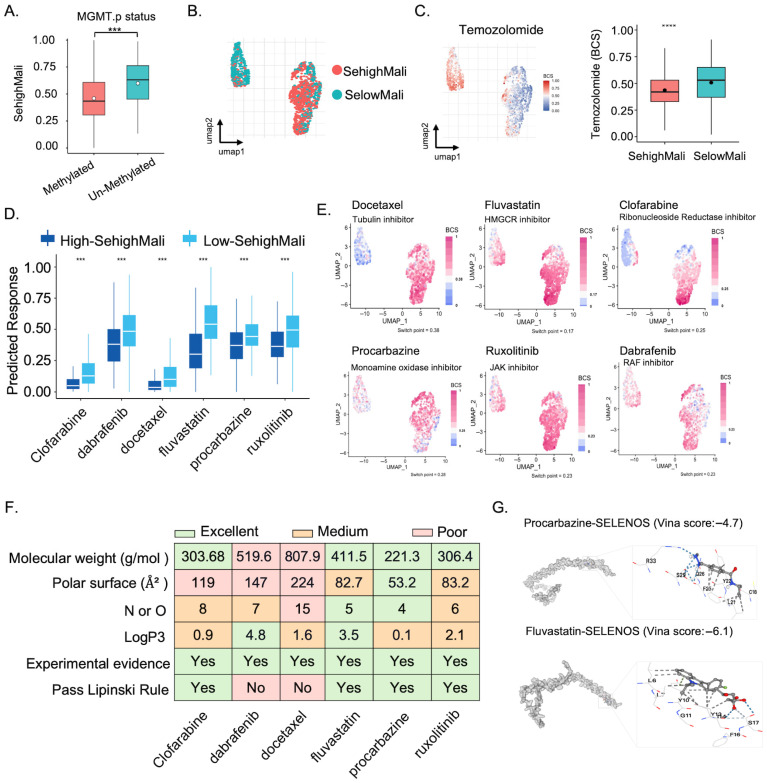
SehighMali-associated drug sensitivity and potential targeting of SELENOS. (**A**) Association between SehighMali abundance and MGMT promoter methylation in TCGA glioma samples. (*** *p* < 0.001). (**B**) UMAP plot of malignant cells colored by SehighMali and SelowMali. (**C**) Left: spatial mapping of predicted temozolomide response (based on BCS score) at single-cell level. Right: SehighMali cells show significantly lower sensitivity to temozolomide compared to SelowMali (**** *p* < 0.0001). (**D**) Predicted drug responses for six compounds across high- and low-SehighMali groups in TCGA dataset. (*** *p* < 0.001) (**E**) Single-cell mapping of predicted drug sensitivity (BCS score) to candidate compounds. (**F**) Physicochemical properties and predicted brain penetration profiles of six candidate compounds. (**G**) Molecular docking simulations showing binding poses of procarbazine and fluvastatin with SELENOS.

Given their clinical relevance, we explored therapeutic vulnerabilities of SehighMali cells. In the TCGA cohort, patients with high SehighMali abundance exhibited lower MGMT promoter methylation (Figure 9A), suggesting resistance to temozolomide (TMZ). Consistently, cell-based drug response prediction at the single-cell level showed that SehighMali cells were significantly less responsive to TMZ than SelowMali cells, supporting a resistant phenotype (Figure 9B,C). To identify alternative therapies, we queried the CTRP dataset and found that clofarabine, dabrafenib, docetaxel, fluvastatin, procarbazine, and ruxolitinib were predicted to be more effective in SehighMali-high samples (Figure 9D). These results were supported at the single-cell level (Figure 9E). Among them, procarbazine and fluvastatin exhibited favorable physicochemical properties, including low molecular weight and compliance with Lipinski’s Rule [45], indicating potential BBB permeability (Figure 9F). Molecular docking further revealed high binding affinity of procarbazine and fluvastatin to SELENOS, suggesting a possible direct interaction (Figure 9G).

## 4. Discussion

In this study, we identified a transcriptionally distinct population of malignant cells, termed SehighMali, characterized by elevated expression of selenoprotein-related genes across bulk, single-cell, and spatial transcriptomic platforms. These cells exhibited enhanced oxidative phosphorylation, MYC pathway activation, elevated DNA repair capacity, and immune-suppressive signaling, and were predominantly enriched in the tumor core. Notably, SehighMali cells preferentially interacted with myeloid cells via the CSF1–CSF1R axis, suggesting their active role in shaping an immunosuppressive tumor microenvironment. The abundance of SehighMali cells was strongly associated with poor prognosis, low MGMT promoter methylation, and predicted resistance to temozolomide, implicating this cellular state as a potential contributor to glioma treatment failure.

Seleno-amino acids and their metabolites have shown significant promise in tumor immunotherapy by modulating immune responses, facilitating tumor–immune cell interactions, and reshaping the tumor microenvironment [46]. Consistent with these findings, we observed that SehighMali cells exhibited prominent immune infiltration-related characteristics. Spatial transcriptomics and bulk RNA-seq analyses consistently demonstrated that SehighMali cells were enriched in regions infiltrated by regulatory T cells (Tregs) and immunosuppressive M2 macrophages, while monocytes and activated NK cells were comparatively depleted. These observations were robustly supported by multiple immune deconvolution methods. Furthermore, patients with high SehighMali displayed marked upregulation of key immune checkpoint molecules, including PDCD1 (PD-1), CD274 (PD-L1), and HAVCR2 (TIM-3), consistent with an immunosuppressive phenotype. Subsequent analyses of cell–cell communication further revealed notably enhanced interactions between SehighMali and myeloid cells relative to SelowMali cells, mediated predominantly via the CSF1/CSF1R signaling axis. It is well-established that CSF1/CSF1R signaling drives the recruitment, survival, and polarization of tumor-associated macrophages toward an immunosuppressive M2 phenotype, facilitating tumor progression and immune evasion [47,48].

Although direct evidence linking selenoproteins to CSF1 expression is currently lacking, we propose several potential mechanisms that may underlie this association. Elevated selenoprotein levels may modulate oxidative stress and endoplasmic reticulum homeostasis, thereby influencing the expression of oncogenic transcription factors such as c-Myc [49]. In addition, SELENOS has been reported to stabilize c-Myc by preventing its ubiquitin–proteasome-mediated degradation in clear cell renal cell carcinoma [50]. Notably, c-Myc has been shown to transcriptionally activate CSF1 and promote the recruitment of immunosuppressive M2-like tumor-associated macrophages in glioblastoma, particularly following radiotherapy [51]. Together, these observations raise the possibility that selenoproteins may contribute to CSF1 expression and the immunosuppressive microenvironment. These findings suggest that targeting selenoprotein biosynthesis may represent a potential strategy to improve the efficacy of glioma immunotherapy. However, the current data do not establish a c-MYC–dependent mechanism, and this relationship should be considered hypothetical and requires further experimental validation.

Among the selenoprotein genes upregulated in SehighMali cells, SELENOS emerged as a candidate contributor to malignant phenotypes. Functional assays demonstrated that SELENOS promotes glioma cell proliferation, invasion, and in vivo tumor growth, underscoring its central role in sustaining the malignant phenotype. Previous studies have implicated SELENOS as a prognostic marker in lower-grade gliomas, where siRNA-mediated knockdown reduced proliferation, viability, and invasiveness of LGG cells [16]. Similarly, we observed that SELENOS knockdown significantly reduced GBM cell proliferation and invasion in vitro and suppressed tumorigenicity in vivo. Our findings not only support the clinical relevance of SELENOS but also highlight its potential as a therapeutic vulnerability. Notably, SehighMali likely reflects a coordinated selenoprotein-enriched program rather than the effect of a single dominant driver, with potential synergistic contributions from multiple selenoproteins, although their individual roles remain to be defined.

Moreover, drug sensitivity predictions based on both bulk and single-cell transcriptomic data revealed that SehighMali cells were selectively sensitive to non-conventional glioma agents, including fluvastatin and procarbazine. Notably, fluvastatin has previously been shown to inhibit proliferation and induce apoptosis in multiple glioblastoma cell lines, alter the malignant phenotype of C6 glioma cells, and enhance the efficacy of chemotherapeutic agents through synergistic interactions [52]. Procarbazine, a component of the PCV regimen (procarbazine, lomustine, vincristine), is currently used as a second-line chemotherapy for glioma [53]. Molecular docking analyses in our study suggested potential interactions between SELENOS and both fluvastatin and procarbazine, suggesting a possible mechanistic basis for drug repurposing. However, the specificity of the predicted drugs and whether they truly modulate the redox-regulating functions of SELENOS require further experimental validation. Moreover, given the essential roles of selenoproteins in physiological redox homeostasis, systemic inhibition may result in significant toxicity. Accordingly, therapeutic strategies may need to prioritize selective or partial modulation and careful evaluation of the therapeutic window.

To our knowledge, this is the first study to systematically characterize the role of selenoproteins in glioma using integrative multi-omic analysis. Our findings demonstrate the utility of combining bulk, single-cell, and spatial transcriptomics to delineate clinically relevant malignant programs in a highly heterogeneous tumor context.

This study has limitations that should be acknowledged. Although in vivo experiments support a functional role for SELENOS, its downstream molecular mechanisms require further elucidation, and whether SELENOS regulates CSF1 expression in a c-MYC-dependent manner requires further investigation. The molecular docking results are based on in silico predictions and do not constitute direct evidence of binding, and therefore require further experimental validation. Moreover, the prognostic and therapeutic significance of SehighMali abundance should be validated in larger clinical cohorts. Finally, due to the inherent spatial resolution constraints of spatial transcriptomics, direct cell–cell interactions cannot be reliably inferred. Consequently, the observed co-localization between SehighMali and myeloid cells may reflect spatial proximity within a shared niche rather than physical cellular contact. These findings require further validation using higher-resolution modalities, such as multiplexed immunohistochemistry (mIHC), in clinical tumor specimens.

## 5. Conclusions

In summary, we identified a selenoprotein-driven malignant glioma cell state, termed SehighMali, characterized by elevated selenoprotein expression, distinct metabolic and immunological features, and enrichment in tumor core regions. SehighMali was associated with aggressive molecular features, poor clinical outcomes, and a predicted temozolomide-resistant phenotype, supporting an important role for selenoprotein-related programs in glioma progression.

We further identified SELENOS as a key regulator of this malignant state. SELENOS knockdown suppressed glioma proliferation, invasion, tumor growth, macrophage recruitment, and CSF1 expression, while promoting macrophage polarization toward a pro-inflammatory phenotype. These findings highlight SELENOS as a potential therapeutic target in glioma.

## Data Availability

The data presented in this study are available on request from the corresponding author.

## Supplementary Materials

The following supporting information can be downloaded at: https://www.mdpi.com/article/10.3390/cancers18091489/s1, Figure S1. Comparison of prognostic models based on selenoprotein genes with published signatures. Figure S2. Prognostic performance of the selenoprotein-based risk score derived from the best-performing machine learning model. Figure S3. Single-cell transcriptomic characterization of selenoprotein-associated programs in glioblastoma. Figure S4. Spatial colocalization of SehighMali and myeloid cells across additional GBM samples. Figure S5. Validation of clinical significance of SehighMali in independent cohorts. Figure S6. Clinical and immunological associations of SelowMali cells in bulk glioma dataset. Figure S7. Uncropped Western blot images related to Figure 7B–D. Table S1. Primers used for qPCR. Tables S2. Pairwise classification consistency across scoring methods.
