# Supplementary material for: Single-Cell and Spatial Transcriptomics Reveals Selenoproteins Shape Immunosuppressive Microenvironment and Therapeutic Outcomes in Glioma
Source: Cancers (Basel). 2026 May 6;18(9):1489. doi: 10.3390/cancers18091489 (PMC13162601; doi:10.3390/cancers18091489)
Supplement: Supplementary file 1 [file cancers-18-01489-s001.zip › cancers-4262292-supplementary.pdf]

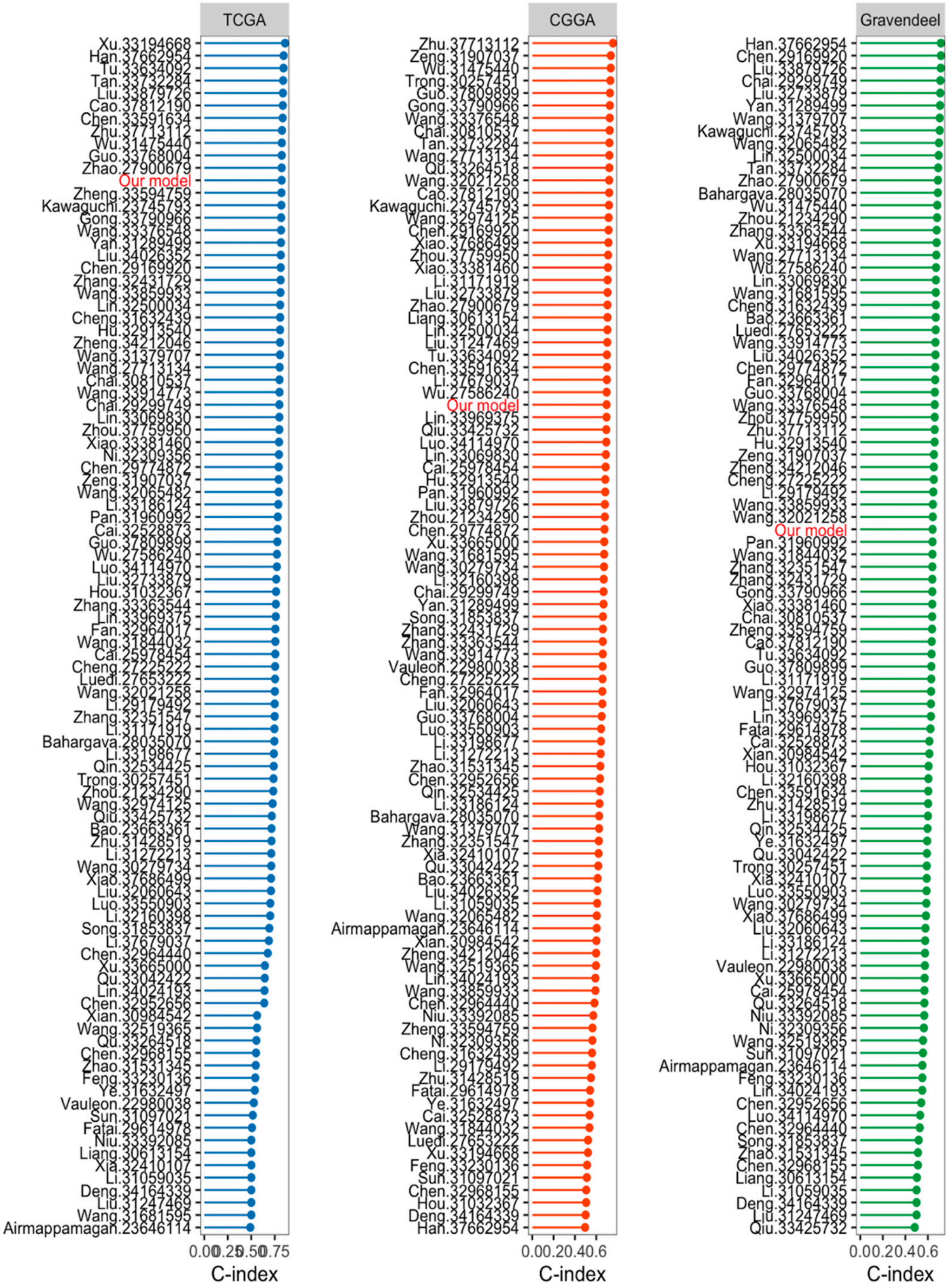

Figure S1. Comparison of prognostic models based on selenoprotein genes with published signatures. C-index of published glioma prognostic signatures evaluated in TCGA, CGGA, and Gravendeel cohorts. The model derived from selenoprotein-related genes (red) exhibits competitive or superior predictive power relative to existing models.

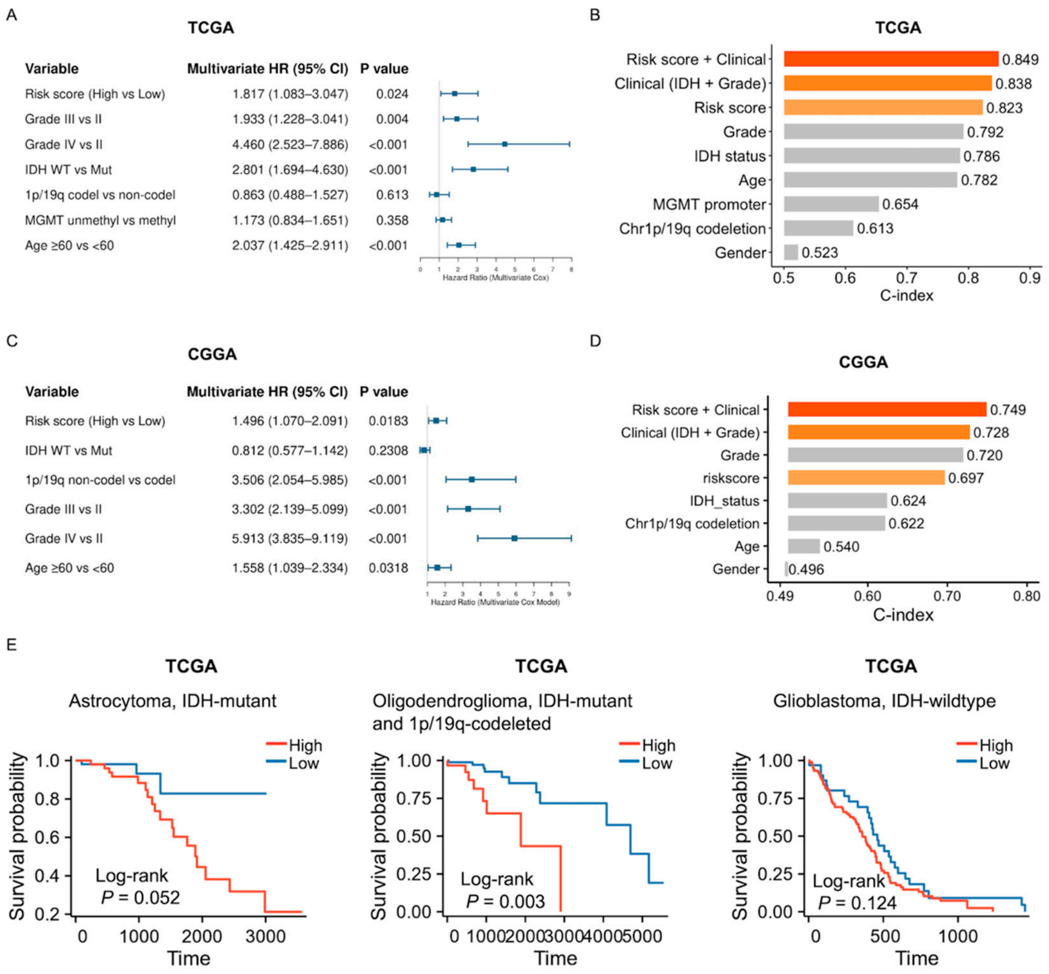

Figure S2. Prognostic performance of the selenoprotein-based risk score derived from the best-performing machine learning model. A. Multivariate Cox regression analysis in the TCGA cohort assessing the independent prognostic value of the selenoprotein-related risk score and clinical variables (grade, IDH status, 1p/19q codeletion, MGMT promoter methylation, and age). B. C-index comparison for the TCGA cohort showing the predictive performance of the risk score, clinical variables, and combined models. C. Multivariate Cox regression analysis in the CGGA cohort evaluating the prognostic significance of the risk score alongside clinical features. D. C-index comparison in the CGGA cohort demonstrating enhanced prognostic accuracy when the risk score is integrated with clinical variables. E. Kaplan–Meier survival curves for TCGA patients stratified by the selenoprotein-related risk score across key molecular subtypes: astrocytoma (IDH-mutant), oligodendroglioma (IDH-mutant and 1p/19q-codeleted), and glioblastoma (IDH-wildtype). Time is shown in days. Log-rank P values are shown, with adjustment for multiple comparisons using the Benjamini–Hochberg method.

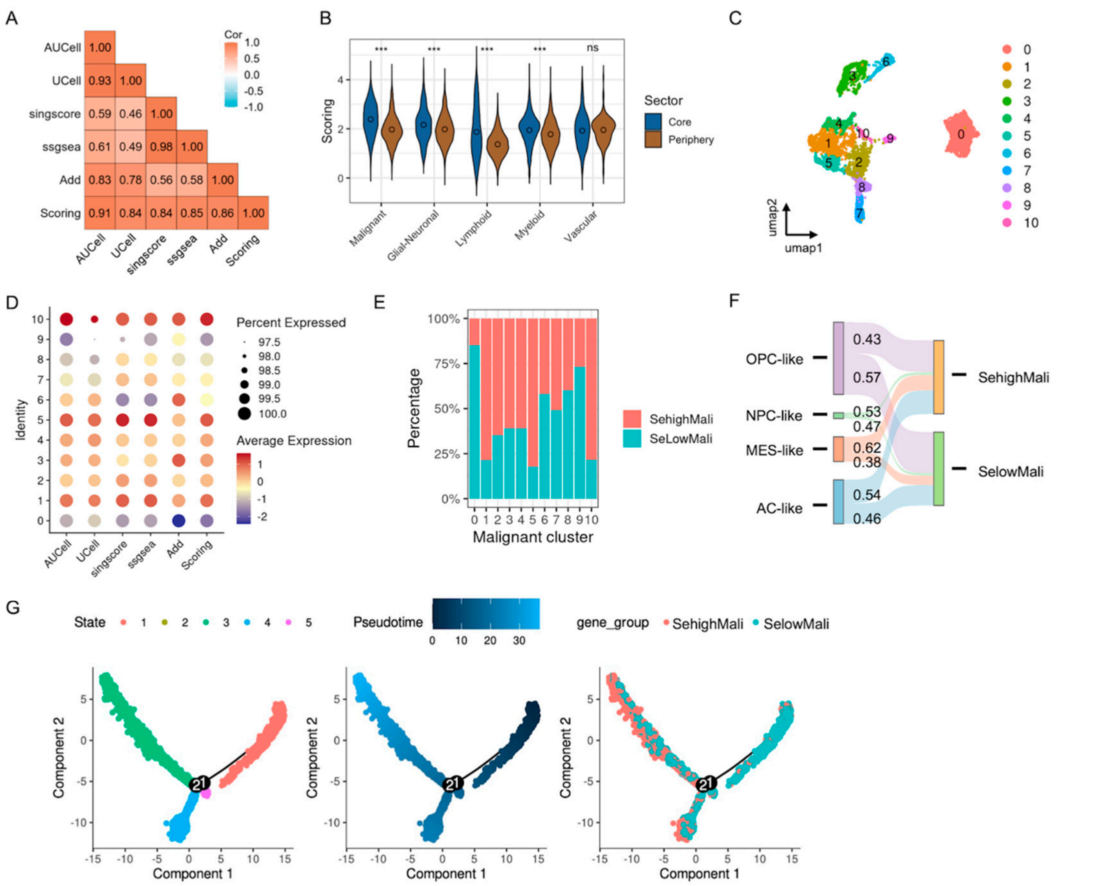

Figure S3. Single-cell transcriptomic characterization of selenoprotein-associated programs in glioblastoma.

A. Heatmap showing Spearman correlation coefficients between six scoring methods (AUCell, UCell, singscore, ssGSEA, AddModuleScore, and Scoring). All pairwise correlations are statistically significant (FDR < 0.001). B. Violin plots show the distribution of selenoprotein scores in different cell types stratified by anatomical sector (core vs. periphery). Scoring was based on the expression of hallmark selenoprotein genes. C. UMAP visualization of identified cell clusters. A Uniform Manifold Approximation and Projection (UMAP) plot shows the dimensionality reduction and clustering of single cells into 11 distinct identities (labeled 0-10), with each cluster represented by a unique color as indicated in the legend. D. Dot plot displaying the expression of selenoprotein (AUCell, UCell, singscore, ssgsea, AddModuleScore, Scoring) across malignant clusters. Dot size represents the percentage of cells expressing the signature, and dot color reflects the averaged expression level. E. Distribution of SehighMali and SeLowMali cell states across malignant clusters. F. Sankey diagram showing the correspondence between canonical malignant cell states (OPC-like, NPC-like, MES-like, AC-like) and the SehighMali and SeLowMali populations. Numbers on each branch represent the fraction of cells contributing to each program. G. Pseudotime trajectory analysis of malignant cells. Cells are visualized along a low-dimensional trajectory and colored by inferred cell states (left), pseudotime (middle), and gene group classification (right; SehighMali vs SelowMali).

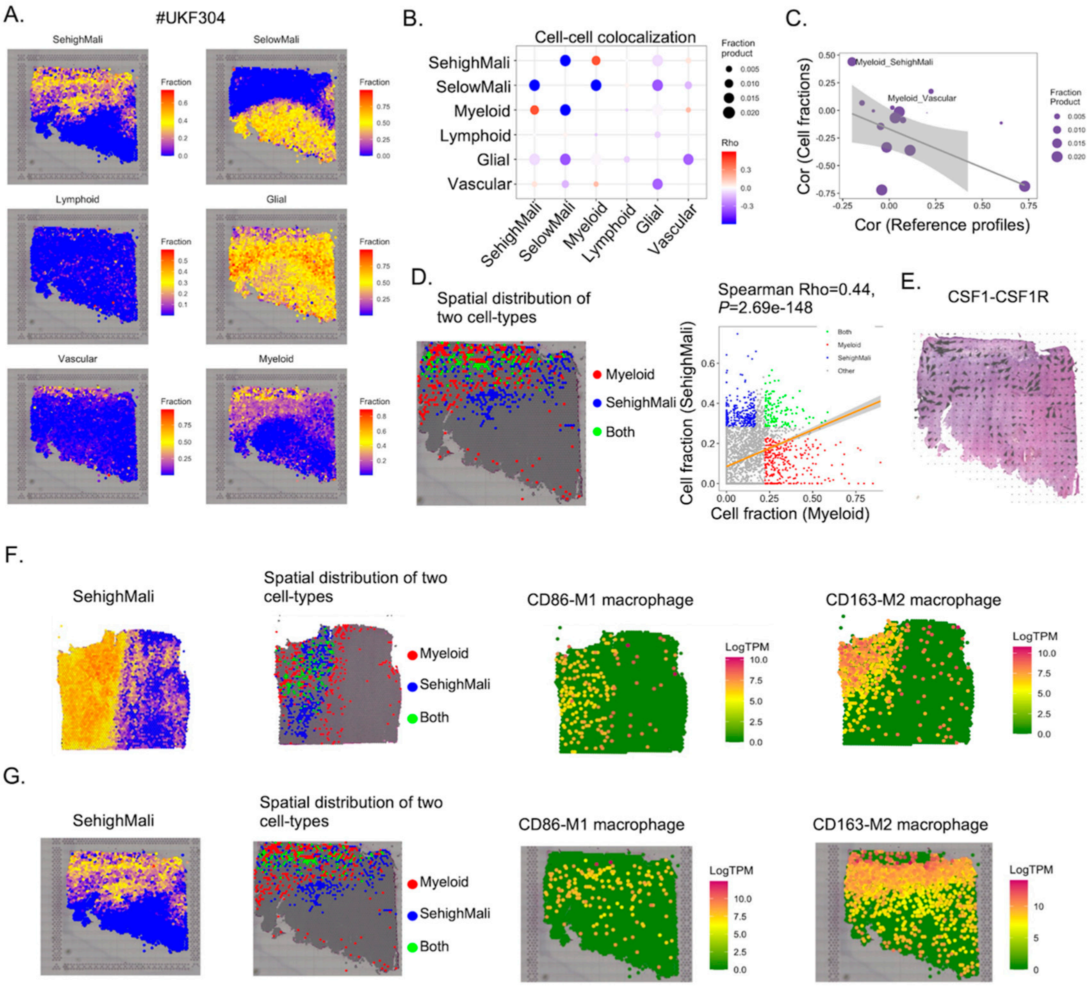

Figure S4. Spatial colocalization of SehighMali and myeloid cells across additional GBM samples.

A. Spatial mapping of key cell types inferred from deconvolution, highlighting SehighMali and SelowMali malignant subgroups, as well as myeloid, lymphoid, vascular, and glial-neuronal compartments for Sample #UKF304. B. Cell–cell colocalization matrix showing spatial proximity patterns across inferred cell types, with strong enrichment between SehighMali and myeloid cells for Sample #UKF304. C. Right: correlation between cell-type colocalization and transcriptomic reference overlap for Sample #UKF304. D. Left: spatial distribution of SehighMali (red) and myeloid (blue) cells shows co-localization within tumor core regions. Right: correlation of SehighMali and myeloid cell fractions across spatial bins ($\rho = 0.44$), supporting tissue-level proximity consistent with enhanced communication observed in transcriptomic data. E. Spatial distribution of CSF1-CSF1R signaling within the GBM sample by COMMOT framework for Sample #UKF304. F-G. Spatial transcriptomic maps showing SehighMali cells (left), their co-distribution with myeloid cells (middle), and the spatial expression of M1 macrophage marker CD86 (right, green) and M2 macrophage marker CD163 (right, green) in sample #UKF269 (F) and #UKF304 (G).

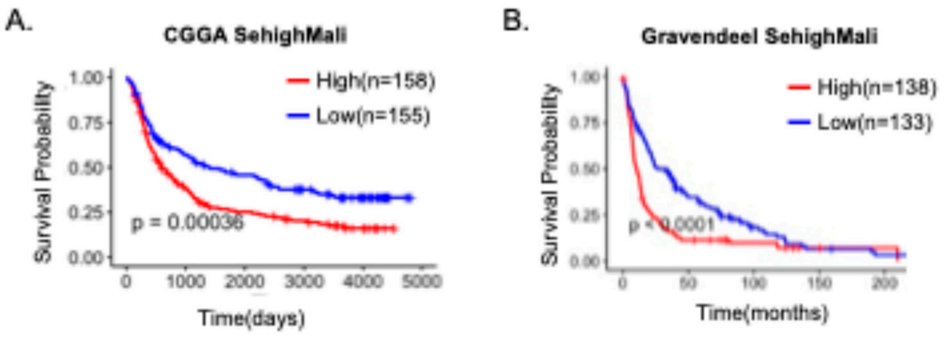

Figure S5. Validation of clinical significance of SehighMali in independent cohorts. A. Kaplan–Meier survival curve comparing overall survival between patients with high (red) and low (blue) SehighMali abundance in CGGA (A) and Gravendeel (B) cohorts. Groups stratified by median value, $P < 0.0001$ (log-rank test).

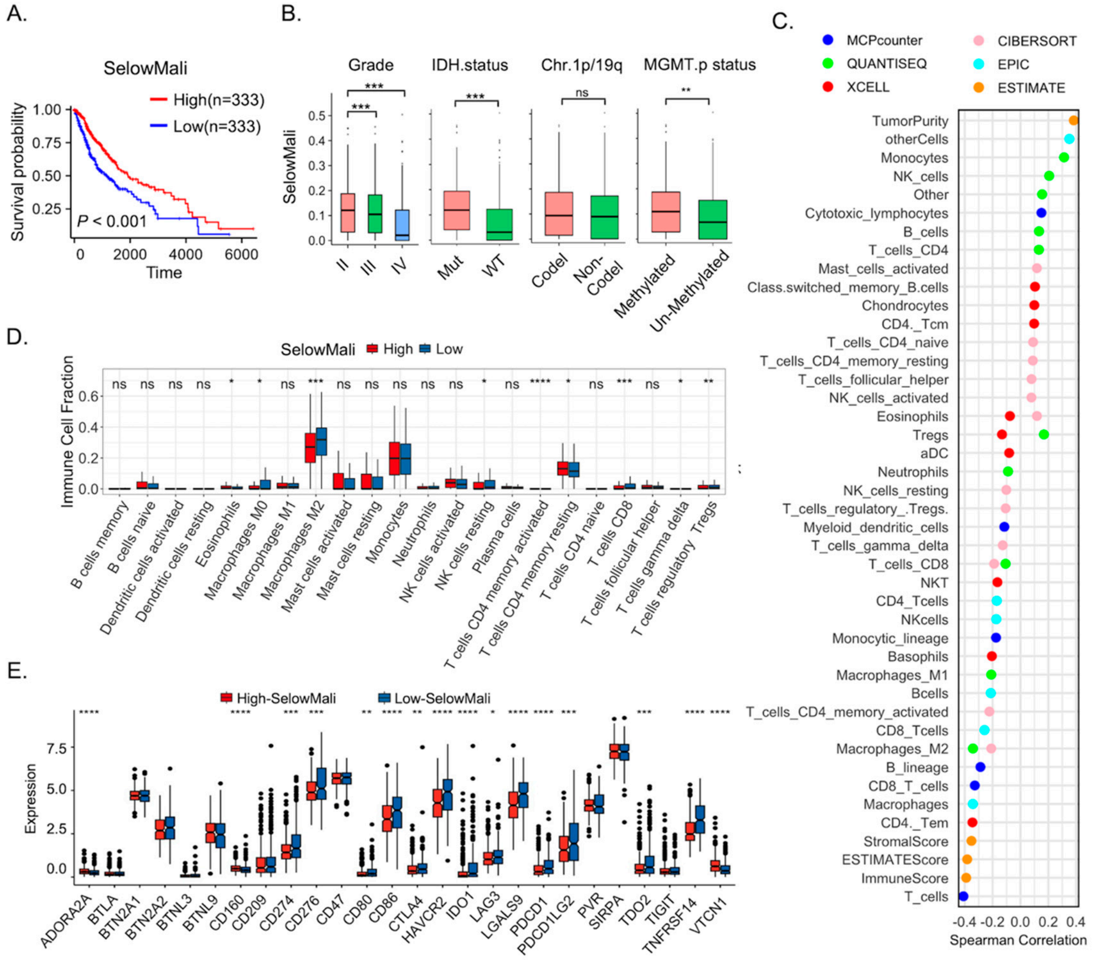

Figure S6. Clinical and immunological associations of SelowMali cells in bulk glioma dataset. A. Kaplan–Meier survival curve comparing overall survival between patients with high and low SelowMali abundance in TCGA cohort. Groups stratified by median value, $P < 0.001$ (log-rank test). B. SelowMali abundance across tumor grade, IDH status, 1p/19q codeletion status and MGMT promoter mutation status. SehighMali is significantly enriched in IDH-wildtype, Grade IV, and non-

codeleted gliomas. ***P<0.001. C. Correlation of SelowMali abundance with immune microenvironment features across six algorithms (MCP-counter, xCell, EPIC, CIBERSORT, QUANTISEQ, ESTIMATE). D. Comparison of immune cell infiltration between high- and low-SelowMali groups based on CIBERSORT deconvolution. (*P<0.05, **P<0.01, ***P<0.001, ****P<0.0001). E. Expression levels of immune checkpoint genes in high- and low-SelowMali groups. (*P<0.05, **P<0.01, ***P<0.001, ****P<0.0001)

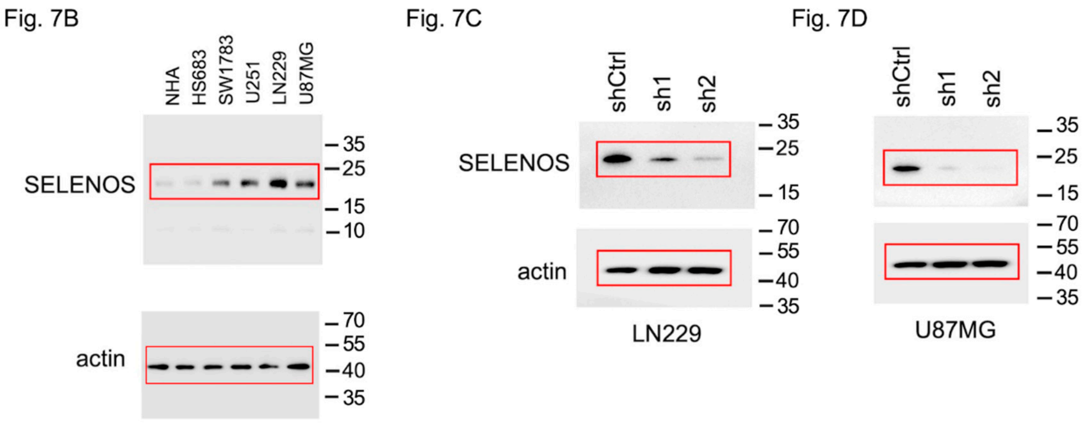

Figure S7. Uncropped Western blot images related to Fig. 7B-D.

Table S1. Primers used for qPCR

Gene	Primer sequences (5’-3’)
GAPDH	Forward: 5’- TGCACCACCAACTGCTTAGC-3’
	Reverse: 5’- GGCATGGACTGTGGTCATGAG-3’
IL-1B	Forward: 5’- ATGATGGCTTATTACAGTGGCAA-3’
	Reverse: 5’- GTCGGAGATTCGTAGCTGGA-3’
IL12B	Forward: 5’- GACATTCTGCGTTCAGGTCCAG-3’
	Reverse: 5’- CATTTTTGCGGCAGATGACCGTG-3’
CD86	Forward: 5’- CCATCAGCTTGTCTGTTTCATTCC -3’
	Reverse: 5’- GCTGTAATCCAAGGAATGTGGTC -3’
ARG1	Forward: 5’- TGATGTTGACGGACTGGACC -3’
	Reverse: 5’- ATCTAATCCTGAGAGTAGCCCTGT -3’
MRC1	Forward: 5’- AGCCAACACCAGCTCCTCAAGA-3’
	Reverse: 5’- CAAAACGCTCGCGCATTGTCCA-3’
CD163	Forward: 5’- CACCTCTTTGGAATGAGCAAACTCAG-3’
	Reverse: 5’- TATTCCTCTGCATGGTTCTTTC-3’

Table S2. Pairwise classification consistency across scoring methods

	AUCell	UCell	singscore	ssgsea	Add	Scoring
AUCell	1	0.885	0.71	0.717	0.83	0.87
UCell	0.885	1	0.66	0.67	0.799	0.816
singscore	0.71	0.66	1	0.945	0.724	0.827
ssgsea	0.717	0.67	0.945	1	0.729	0.836
Add	0.83	0.799	0.724	0.729	1	0.853
Scoring	0.87	0.816	0.827	0.836	0.853	1
